# Muscle quality, physical performance, and comorbidity are predicted by circulating procollagen type III N-terminal peptide (P3NP): the InCHIANTI follow-up study

**DOI:** 10.1007/s11357-023-00894-3

**Published:** 2023-08-02

**Authors:** Raffaello Pellegrino, Roberto Paganelli, Angelo Di Iorio, Stefania Bandinelli, Antimo Moretti, Giovanni Iolascon, Eleonora Sparvieri, Domiziano Tarantino, Luigi Ferrucci

**Affiliations:** 1 Department of Scientific Research, Campus Ludes, Off-Campus Semmelweis University, 6912, Pazzallo, Lugano, Switzerland; 2 Saint Camillus International, University of Health and Medical Sciences, Rome, Italy; 3 Department of Innovative Technologies in Medicine & Dentistry, University “G. d’Annunzio”, 66100 Chieti-Pescara, Italy; 4 Geriatric Unit, Azienda Toscana Centro, Florence, Italy; 5 Department of Medical and Surgical Specialties and Dentistry, University of Campania “Luigi Vanvitelli”, 80138 Naples, Italy; 6 Department of Internal Medicine, ASL Teramo, Teramo, Italy; 7 Department of Public Health, University of Naples Federico II, 80131 Naples, Italy; 8 Longitudinal Studies Section, Translational Gerontology Branch, National Institute On Aging, National Institutes of Health, Baltimore, MD 21224 USA

**Keywords:** P3NP, Skeletal muscle function deficit, Adipokines, Short Physical Performance Battery, Longitudinal study

## Abstract

Sarcopenia is characterized by skeletal muscle quantitative and qualitative alterations. A marker of collagen turnover, procollagen type III N-terminal peptide (P3NP), seems to be related to those conditions. This study aims to assess the predictive role of P3NP in muscle density and physical performance changes. In the InCHIANTI study, a representative sample from the registry lists of two towns in Tuscany, Italy, was recruited. Baseline data was collected in 1998, and follow-up visits were conducted every 3 years. Out of the 1453 participants enrolled at baseline, this study includes 1052 participants. According to P3NP median levels, population was clustered in two groups; 544 (51.7%) of the 1052 subjects included were classified in the low median levels (LM-P3NP); at the baseline, they were younger, had higher muscle density, and performed better at the Short Physical Performance Battery (SPPB), compared to the high-median group (HM-P3NP).

LM-P3NP cases showed a lower risk to develop liver chronic diseases, CHF, myocardial infarction, and osteoarthritis. HM-P3NP levels were associated with a longitudinal reduction of muscle density, and this effect was potentiated by the interaction between P3NP and leptin. Moreover, variation in physical performance was inversely associated with high level of P3NP, and directly associated with high fat mass, and with the interaction between P3NP and muscle density. Our data indicate that P3NP is associated with the aging process, affecting body composition, physical performance, and clinical manifestations of chronic degenerative age-related diseases.

**Supplementary Information:**

The online version contains supplementary material available at 10.1007/s11357-023-00894-3.

## Introduction

The decline of muscle mass and function, known as sarcopenia, is closely linked to impaired physical function, frailty, disability, and an increased risk of mortality in the aging population [1]. Apart from the established age-related quantitative loss of muscle tissue, much less is known about muscle quality impairment with aging, namely, the accumulation of fat and connective tissues in skeletal muscle that can contribute to significant mobility impairment with aging [2, 3]. Myosteatosis is characterized by the infiltration of adipose tissue at both intramuscular and intermuscular levels, and it has been proposed that intramyocellular fat deposition can also affect glucose metabolism [4, 5]. Therefore, in the context of age-related muscle wasting, myosteatosis is a potentially crucial aspect of muscle composition and physiology, affecting gait pattern through metabolic and functional alterations in skeletal muscle [4]. Myosteatosis might contribute to the negative effects of obesity and diabetes by interfering with energy metabolism, insulin sensitivity, and calcium uptake in skeletal muscles [6]. Despite growing interest among researchers, the pathophysiology of age-related myosteatosis in humans remains largely unknown. Most hypotheses are based on animal studies or small cross-sectional studies [7], demonstrating that alterations in fatty acid transport, uptake and storage, musculoskeletal mitochondrial activity, muscle stem cell impairment, and infiltration of white cellular elements with the contextual release of adipokines may occur in aging muscles. Aging of skeletal muscle is also associated with myofibrosis that is characterized by an increase in fibrous connective tissue, with a less adequate regenerative capacity of the muscle that is replaced by fibrous connective tissue [8]. Also for myofibrosis, the pathophysiology in humans remains largely unknown. Higher levels of myofibrosis are strictly associated with a higher perimuscular subcutaneous adipose tissue, and with a consequent higher level of chronic low-grade inflammation; altogether those data suggest a cross-talk between the two tissues that could be also involved in the age-related variation in physical performance [9].

An unmet need in this research field is defining quantifiable muscle quality parameters, such as instrumental or biochemical biomarkers. Recent studies have suggested that the circulating procollagen type III N-terminal peptide (P3NP) may be associated with sarcopenia [10] and physical function impairment [11, 12]. These findings could potentially lead to new diagnostic and therapeutic approaches for age-related muscle disorders. P3NP is an essential component of collagen, specifically the amino-terminal propeptides. It is cleaved by specific procollagen proteinases, but not entirely, during collagen maturation. As a result, it is released into the bloodstream during collagen fiber degradation [13]. P3NP is a reliable marker of systemic active fibrosis and reflects muscle remodeling with great accuracy [14]. Studies have suggested that P3NP levels increase in chronic liver diseases, lung diseases, and cardiac diseases such as ischemic heart disease and congestive heart failure (CHF) [13]. These clinical conditions are all involved in age-related physical performance decline.

Our hypothesis is that P3NP, a collagen turnover biomarker, could be a link between disease-related fibrosis, age-related changes in muscle properties, and consequently as an indicator of physical performance reduction in the aging process.

The primary aim of this study is to better understand the role of P3NP in the genesis of diseases and comorbidities, how those clinical events could mediate the age-related skeletal muscle density variation, and lastly how those markers predict the age-related decline of physical performance, in a large and representative Italian population, from the InCHIANTI study.

## Methods

The design of the InCHIANTI study has been described in detail elsewhere [15]. Briefly, the study was designed by the Laboratory of Clinical Epidemiology of the Italian National Institute of Research and Care on Aging (INRCA, Florence, Italy), and was performed in two small towns in Tuscany. The baseline data were collected in 1998–2000; follow-up took place every 3 years (Supplementary Fig. 1).

### Samples

Of the 1453 participants enrolled at baseline in the InCHIANTI study, 1052 subjects were included in this study and had at least one baseline assessment, donated a blood sample, and had all variables of interest for this study. Participants were all European subjects and of Caucasian race. The Ethical Committee of the Local Health Authority of Florence, Tuscany region, approved the study protocol, and written informed consent was obtained from each participant.

### Tibial pQCT

The peripheral quantitative computed tomography (pQCT) was performed using the XCT 2000 device (Stratec Medizintechnik, Pforzheim, Germany). A detailed description of the tibial QCT examination, in the InCHIANTI study, has been published elsewhere [16]. The images obtained from the pQCT were analyzed using the BonAlyse software (BonAlyse Oy, Jyvaskyla, Finland). Using pQCT images obtained at 66% of the tibia length from the distal tip of the tibia, we estimated muscle density and the bone-to-muscle ratio both expressed in mg/cm^3^. Lastly, from the same scan performed at 66% of the tibial length, the maximal cross-sectional fat area (subcutaneous and intramuscular adipose tissue) [17] was calculated, and expressed in mm^2^.

### Laboratory tests

Fasting blood samples were collected in the morning after a 12-h fast, centrifuged, and stored at – 80 °C. Insulin was measured in serum samples stored at – 80 °C. Serum glucose was assessed using the hexokinase method (Boehringer Mannheim, Mannheim, Germany). Serum insulin levels were quantified using the Cobas Roche electrochemiluminescence immunoassay (12017547 122) on a Modular Analytics E170 analyzer (Roche Diagnostics). The interassay CV was < 4.9% for insulin and 1.7% for glucose.

Serum leptin was determined using ELISA (Human Endocrine LINCOplex Kit; MDC = 1 ng/mL in 100 μL sample, CV < 7%).

Ghrelin was analyzed using a commercial kit (Human Ghrelin ELISA kit, Phoenix Pharmaceuticals, Inc, Belmont, CA, USA). Measures were expressed in ng/mL with an intra-assay CV of 5% and inter-assay < 14% and range of 0.1–100 ng/mL.

Serum adiponectin concentration was measured using RIA assay (Human Adiponectin RIA Kit; LINCO Research, Inc, MO, USA), and the measure unit was expressed in nanograms per milliliter. For adiponectin, the MDC was 1 ng/mL in a 100 μL sample, and the intra-assay CV was < 7% and inter-assay CV was < 10%.

Serum intact N-terminal propeptide of type III procollagen (P3NP) was measured using radioimmunoassay (PIIINP RIA, Immunodiagnostic Systems, Fountain Hills, AZ, USA); the measure unit was expressed in nanograms per milliliter. The intra-assay CV was 14.5%, and inter-assay CV was 17.3%.

### Diseases and comorbidities

Based on self-report, the diagnosis of major medical condition was ascertained according to preestablished criteria that combine reported doctor diagnosis eventually supported by medical records, physical examination, blood tests, and drug prescription [18]. Comorbidity score was calculated summing the number of diseases reported at every follow-up visit.

### Physical performance and strength

The Short Physical Performance Battery (SPPB) is a tool used to assess lower extremity performance, based on the Established Populations for the Epidemiologic Studies of the Elderly (EPESE) [19]. SPPB consists of three tests: walking speed, ability to stand from a chair, and ability to maintain balance in progressively more challenging positions. Each test is scored on a five-level scale, with 0 indicating inability to perform the test and 4 indicating the highest level of performance. The scores from the three tests are then added together to create a summary physical performance measure, ranging from 0 (worst) to 12 (best). In addition to the SPPB, handgrip strength was also measured using a handheld dynamometer (hydraulic hand “BASELINE”; Smith & Nephew, Agrate Brianza, Milan, Italy). Participants were asked to perform the task twice with each hand, and the average of the best result obtained with each hand was used for analysis.

### Statistical analysis

According to P3NP median levels (4.021; Q1–Q3 3.290–4.821), the population was clustered into two groups: high (HM-P3NP) and low (LM-P3NP). Baseline characteristics were compared between P3NP groups for all variables of interest using analysis of variance for continuous variables and *χ*^2^ test analyses for dichotomous or categorical variables. Moreover, in the descriptive table, we reported the *p* values adjusted for age and sex, using linear and logistic regression models, respectively. To investigate the P3NP predictive risk in baseline and follow-up diseases reporting, generalized estimating equation (GEE) models were used to specify a repeated measure model with a dichotomous outcome variable (binary logistic regression) and multiple independent factors [20]. GEE repeated measure model, with a Poisson distribution, was also used to assess the association between P3NP groups and the comorbidity score variation across time of the study [20]. To test whether circulating levels of P3NP at baseline were associated with changes in muscle density and with SPPB, we performed linear mixed-effect models with each parameter as the dependent variable and baseline P3NP and its interaction with follow-up time (years) as the independent variable. Our models included the following fixed effects: all variables at univariate analysis showed a *p* value < 0.10, and all diseases statistically significant associated with P3NP (liver chronic diseases, CHF, myocardial infarction, and osteoarthritis); and random effects: intercept and slope, with unstructured covariance. SAS version 9.4 for Windows (SAS Institute, Inc., Cary, NC) was used for all data processing and statistical analyses. We set the level of statistical significance at *p* < 0.05 (two-sided).

## Results

Five hundred and forty-four (51.7%) of the 1052 included subjects were classified in the LM-P3NP level group; at the baseline, the same group was younger (*p* value = 0.005), had higher muscle density (*p* value < 0.001), and performed better at the SPPB (*p* value = 0.001), compared to the HM-P3NP level group (Table 1). Moreover, in the HM-P3NP, serum insulin (*p* value < 0.001), adiponectin (*p* value < 0.001), and leptin (*p* value = 0.005) showed statistically significant higher serum levels compared to LM-P3NP.

**Table 1:** Body composition, functional, and laboratory characteristics in the InCHIANTI population study at baseline, according to upper and lower median procollagen type III N-terminal peptide (P3NP) levels. All *p* values were age and sex adjusted

	Lower median P3NP levels	Upper median P3NP levels	*p* value*
	544	508	
Age (years)	65.26 ± 15.51	69.25 ± 15.56	0.001
Female sex (no.)	311 (57.11)	283 (55.71)	0.63
Muscle area at 66% tibia length (cm^2^)	63.67 ± 13.02	63.75 ± 13.46	0.20
Muscle density at 66% tibia length (mg/cm^3^)	71.92 ± 3.34	70.74 ± 4.03	< 0.001
Fat area at 66% tibia length (cm^2^)	12.23 ± 7.61	12.04 ± 8.21	0.32
Bone to muscle ratio at 66% tibia length (mg/cm^3^)	10.20 ± 2.72	10.44 ± 3.80	0.51
Handgrip (kg)	33.13 ± 14.03	30.21 ± 14.04	0.06
SPPB score (0–12)	10.90 ± 1.99	10.15 ± 2.82	0.001
P3NP (µg/L)	3.17 ± 0.63	5.32 ± 1.68	< 0.001
Adiponectin (µg/mL)	12.01 ± 8.94	14.97 ± 10.61	< 0.001
Plasma insulin (mIU/L)	10.01 ± 5.33	11.76 ± 7.15	< 0.001
Leptin (ng/mL)	11.94 ± 16.06	14.55 ± 15.86	0.005
Ghrelin (ng/mL)	0.28 ± 0.05	0.28 ± 0.05	0.68

*p value is age and sex adjusted

### Disease diagnosis

Upon examining the cross-sectional distribution of diseases, we found that individuals with HM-P3NP levels were more likely to report myocardial infarction (OR 2.34; 95% CI 1.25–4.38; *p* value = 0.001), angina pectoris (OR 1.68; 95% CI 1.09–2.59; *p* value = 0.009), chronic liver disease (OR 2.13; 95% CI 1.17–3.89; *p* value = 0.004), osteoarthritis (OR 1.39; 95% CI 1.03–1.86; *p* value = 0.009), and CHF (OR 1.58; 95% CI 1.16–2.15; *p* value = 0.001). These findings suggested a potential correlation between elevated P3NP levels and the prevalence of these conditions (Table 2).

**Table 2:** Distribution of diseases found at baseline in the InCHIANTI study according to lower and upper median procollagen type III N-terminal peptide (P3NP) levels. In the table were reported the *p* value for the differences between the two study groups and the OR and 95% CI; unadjusted *p* value is reported

	Lower median P3NP levels	Upper median P3NP levels	*p* value	OR (95% CI)
	544	508		
Myocardial infarction	14 (2.57)	36 (7.09)	0.001	2.34 (1.25–4.38)
Angina pectoris	33 (6.07)	58 (11.42)	0.009	1.68 (1.09–2.59)
Chronic liver disease	14 (2.57)	28 (5.51)	0.004	2.13 (1.17–3.89)
Hypertension	313 (57.54)	313 (61.61)	0.37	0.86 (0.64–1.15)
Osteoarthritis	114 (20.96)	148 (29.13)	0.009	1.39 (1.03–1.86)
Diabetes	52 (9.56)	59 (11.61)	0.46	1.35 (0.93–1.96)
Peripheral arterial disease	71 (13.05)	88 (17.32)	0.12	1.08 (0.77–1.51)
Stroke	21 (3.86)	27 (5.31)	0.43	1.52 (0.89–2.58)
Congestive heart failure	87 (15.99)	139 (27.36)	< 0.001	1.58 (1.16–2.15)

A repeated measure model was used to evaluate the risk of developing major diseases, based on P3NP levels, using GEEs with a binary distribution (Table 3). The results showed that individuals in the HM-P3NP group had a higher likelihood of being diagnosed with chronic liver disease (IRR 1.88; 95% CI 1.02–3.45), CHF (IRR 1.70; 95% CI 1.19–2.42), myocardial infarction (IRR 1.92; 95% CI 1.02–3.59), and osteoarthritis (IRR 1.48; 95% CI 1.04–2.10), compared to those in the LM-P3NP group, regardless of age and time of diagnosis. However, there was no significant second-order effect for the interaction between P3NP levels and disease occurrence, indicating that P3NP levels were not a reliable predictor of disease development. It is worth noting that there were no differences in the diagnosis of stroke, peripheral artery disease, diabetes, hypertension, and angina between the two groups.

**Table 3:** Generalized estimating equation (GEE) logistic regression for repeated measures, factors associated with disease diagnosis. Upper median procollagen type III N-terminal peptide (P3NP) and female sex are the reference groups

	LM-P3NP	Time	P3NP × time	Age	Sex
	IRR (95% CI)	IRR (95% CI)	IRR (95% CI)	IRR (95% CI)	IRR (95% CI)
Liver chronic disease	0.53 (0.28–0.99)	1.28 (1.17–1.40)	0.93 (0.81–1.07)	1.00 (0.99–1.01)	0.80 (0.48–1.32)
Congestive heart failure	0.59 (0.41–0.84)	1.51 (1.41–1.63)	1.13 (1.02–1.26)	1.06 (1.04–1.07)	0.72 (0.56–0.91)
Stroke	0.70 (0.38–1.30)	1.30 (1.17–1.44)	1.11 (0.95–1.30)	1.09 (1.07–1.11)	0.61 (0.40–0.94)
Peripheral arterial disease	0.83 (0.54–1.28)	1.40 (1.29–1.51)	1.06 (0.94–1.18)	1.05 (1.04–1.07)	0.60 (0.46–0.79)
Diabetes	0.82 (0.53–1.26)	1.24 (1.15–1.33)	0.99 (0.89–1.09)	1.03 (1.02–1.04)	0.82 (0.60–1.13)
Osteoarthritis	0.68 (0.47–0.96)	1.65 (1.52–1.78)	1.05 (0.94–1.17)	1.04 (1.03–1.05)	2.55 (2.01–3.23)
Hypertension	1.10 (0.81–1.48)	1.40 (1.30–1.51)	1.00 (0.90–1.10)	1.06 (1.05–1.07)	1.01 (0.79–1.30)
Angina pectoris	0.65 (0.40–1.06)	1.22 (1.13–1.31)	1.09 (0.97–1.23)	1.08 (1.06–1.09)	0.72 (0.51–1.03)
Myocardial infarction	0.52 (0.28–0.98)	1.45 (1.31–1.62)	1.16 (0.99–1.37)	1.04 (1.03–1.05)	0.59 (0.41–0.85)

### Total comorbidity

A GEE logistic regression for repeated measures was conducted to evaluate the predictive value of LM-P3NP for total comorbidity at every time point of the study, with a Poisson distribution. The results showed that the LM-P3NP group had a lower total comorbidity compared to the HM-P3NP group (IRR 0.82; 95% CI 0.74–0.90, *p* value < 0.001), regardless of age (IRR 1.02; 95% CI 1.01–1.03, *p* value < 0.001) and sex (*p* value = 0.26). Additionally, a statistically significant interaction was observed between time and P3NP level, indicating that the comorbidity score increased for both P3NP groups, but with a different time rate, across the different follow-up times (IRR; 95% CI 1.04; 1.02–1.06, *p* value = 0.002). Furthermore, in the model, the death status was also considered, to assess its potential competitive role, and it showed a statistically significant association with the outcome (IRR 1.20; 95% CI 1.09–1.32, *p* value < 0.001).

### Muscle density

The mean muscle density across individuals and time was 70.69 ± 0.09 (mg/cm^3^). Within-person variance was 5.48 ± 0.16 (mg/cm^3^), while between-person variance was 9.20 ± 0.47 (mg/cm^3^). Differences among subjects accounted for approximately 63% of the total variation in muscle density (Table 4; model A). When the time effect was considered, a mean reduction of − 0.41 ± 0.04 (mg/cm^3^) was observed for each follow-up. However, there was still a 10.39 ± 0.89 (mg/cm^3^) unexplained within-person residual variance, indicating that approximately 10% of the within-person variation in muscle density was associated with time (Table 4; model B). The fully adjusted model considered all variables that showed a *p* value < 0.10 in the comparison between median-level groups in the univariate analysis. The parsimonious model (model C) included only P3NP, leptin, fat mass, osteoarthritis, age, and sex that were all associated with variation of muscle density in the study times. Interestingly, the second-order interaction analysis reported in model D revealed that while muscle density changed in a statistically significant manner across time (− 0.54 ± 0.06 mg/cm^3^), the interaction between time and P3NP did not reach a clear statistical significance (*p* value = 0.07). However, a multiplicative effect was observed for the interaction between P3NP and serum level of leptin (− 0.04 ± 0.01, *p* value < 0.001) regardless of age, sex, fat mass, and osteoarthritis.

**Table 4:** Linear mixed model: analysis of variation of muscle density during the study times according to procollagen type III N-terminal peptide (P3NP) median levels

			Model A Unconditional means model	Model B Unconditional growth model	Model C Person-level covariate	Model D Interaction fully adjusted
Initial status	Intercept	*γ*_00_	70.69 ± 0.09***	71.55 ± 0.13***	79.88 ± 0.48***	79.73 ± 0.49***
	P3NP	*γ*_01_			− 0.62 ± 0.17***	− 0.36 ± 0.29
	Age	*γ*_02_			− 0.11 ± 0.01***	− 0.11 ± 0.01***
	Sex	*γ*_03_			− 1.08 ± 0.21***	1.09 ± 0.20***
	Leptin	*γ*_04_			− 0.03 ± 0.01***	− 0.01 ± 0.01
	Fat mass	*γ*_05_			− 0.001 ± 0.0001***	− 0.001 ± 0.000***
	Arthrosis	*γ*_06_			− 1.15 ± 0.21***	− 1.15 ± 0.21***
	Leptin × P3NP	*γ*_01 × 04_				− 0.04 ± 0.01***
Rate of change	Intercept (time)	*γ*_10_		− 0.41 ± 0.04***	− 0.48 ± 0.04***	− 0.54 ± 0.06***
	Time × P3NP	*γ*_11_				0.14 ± 0.08
Level 1	Within person	*δ*^2^_e_	5.48 ± 0.16***	4.93 ± 0.19***	4.93 ± 0.20***	4.94 ± 0.20***
Level 2	In initial status	*δ*^2^_0_	9.20 ± 0.47***	10.39 ± 0.89***	6.44 ± 0.81***	6.35 ± 0.81***
	In rate of change	*δ*^2^_1_		0.15 ± 0.08*	0.19 ± 0.09*	0.18 ± 0.09*
	Covariance	*δ*_01_		− 0.34 ± 0.24	− 0.43 ± 0.25	− 0.43 ± 0.24

*p value < 0.05; **p value < 0.01; ***p value < 0.001

### SPPB

The mean score for the SPPB was found to be 9.42 ± 0.09 across individuals and time. Within-person variance was calculated to be 3.15 ± 0.09, while between-person variance was 9.40 ± 0.43. It was estimated that 75% of the total variation in muscle density could be attributed to differences between subjects (Table 5; model A). When the time effect was considered in the analysis, a mean reduction of − 0.80 ± 0.04 was observed for each follow-up. However, there was still 5.92 ± 0.43 unexplained within-person residual variance. Approximately 54% of the within-person variation in muscle density was associated with time (Table 5; model B). In model C, the parsimonious model was reported with all the variables that were associated with SPPB variation across the study’s timeline. In model D, the fully adjusted interaction model was reported. Interestingly, no interaction was found for time. However, a multiplicative effect was found for the interaction between P3NP for leptin (− 0.02 ± 0.01 *p* value = 0.04) and for P3NP for muscle density (0.07 ± 0.02, *p* value = 0.002). The model explained almost 23% of the variation in the initial status.

**Table 5:** Linear mixed model: analysis of variation of Short Physical Performance Battery during the study times according to procollagen type III N-terminal peptide (P3NP) median levels

			Model A Unconditional means model	Model B Unconditional growth model	Model C Person-level covariate	Model D Interaction fully adjusted
Initial status	Intercept	*γ*_00_	9.42 ± 0.09***	10.88 ± 0.08***	7.28 ± 1.12***	9.57 ± 1.33***
	P3NP	*γ*_01_			− 0.25 ± 0.12*	− 4.85 ± 1.60**
	Age	*γ*_02_			− 0.04 ± 0.01***	− 0.04 ± 0.01***
	Sex	*γ*_03_			− 0.37 ± 0.18*	− 0.40 ± 0.18*
	Leptin	*γ*_04_			− 0.01 ± 0.005**	− 0.001 ± 0.008
	Fat mass	*γ*_05_			0.04 ± 0.01***	0.04 ± 0.01***
	Adiponectin	*γ*_06_			− 0.02 ± 0.01*	− 0.02 ± 0.01*
	Muscle density	*γ*_07_			0.09 ± 0.01***	0.05 ± 0.02***
	Handgrip	*γ*_08_			0.02 ± 0.01***	0.03 ± 0.01***
	P3NP × leptin	*γ*_01 × 04_				− 0.02 ± 0.01*
	P3NP × muscle density	*γ*_01 × 07_				0.07 ± 0.02**
Rate of change	Intercept (time)	*γ*_10_		− 0.80 ± 0.04***	− 0.34 ± 0.03***	− 0.35 ± 0.03***
Level 1	Within person	*δ*^2^_e_	3.15 ± 0.09***	1.45 ± 0.06***	1.13 ± 0.06***	1.12 ± 0.06***
Level 2	In initial status	*δ*^2^_0_	9.40 ± 0.43***	5.92 ± 0.43***	3.14 ± 0.35**	3.08 ± 0.34***
	In rate of change	*δ*^2^_1_		0.97 ± 0.08***	0.32 ± 0.05***	0.32 ± 0.05***
	Covariance	*δ*_01_		0.64 ± 0.14***	− 0.32 ± 0.12***	− 0.32 ± 0.12**

*p value < 0.05; **p value < 0.01; ***p value < 0.001

## Discussion

Our study addresses the question whether serum P3NP levels are associated with qualitative skeletal muscle parameters, physical performance, and comorbidity occurrence in aging people. Our findings demonstrate that HM-P3NP levels are associated with a reduction of muscle density, and this effect is potentiated by the interaction between P3NP and leptin; moreover, variation in physical performance is inversely associated with high level of P3NP, and directly associated with the interaction between P3NP and muscle density. The HM-P3NP group showed a higher risk to develop liver chronic diseases, CHF, myocardial infarction, and osteoarthritis, and finally, the number of comorbidities found at different study follow-up times was significantly higher in the HM-P3NP group. Altogether, these data show that P3NP is clearly associated with the aging process, affecting body composition, physical performance, and clinical manifestation of age-related diseases.

In the Long Life Family (LLF) longitudinal study, P3NP levels were inversely associated with physical performance. Specifically, a decrease in SPPB total score, gait speed, and grip strength were observed in patients with higher P3NP levels [11]. Furthermore, in a cross-sectional study (the Framingham Offspring Study), an inverse association between plasma P3NP levels and total and appendicular lean mass in women was found [21].

Our study, consistently with the outcomes of the LLF study, supports the association between high levels of P3NP and a reduction in physical performance over a long follow-up period. However, we also found a multiplicative effect for the interaction between HM-P3NP levels and muscle density, presumably related to improved physical performance. Moreover compared to LLF cohort, our results consider several confounders that were not analyzed in the work of Santanasto et al., for example, the role of new disease occurrence in the times of the study, that in the LLF cohort were not considered, and analyzed only cross-sectional, and the effect of adipose tissue metabolism in the physical performance variation over time, predicted by P3NP. Diseases and adipose tissue were both considered as promoters of fibrosis in the aging processes [22].

Fibrosis is not a uniform process, but rather a complex entity with multiple mechanisms that can lead to different outcomes and clinical phenotypes [23, 24]. In our study, these seemingly contradictory results may represent two phases of the P3NP cycle, fibrosis, and anabolic phase. P3NP can be released into the bloodstream during two different moments: as a result of cleavage by specific proteinases during the anabolic phase and during fibrillary collagen turnover in the catabolic phase [13]. Therefore, the results that identify the association between high levels of P3NP and muscle density could reflect the pro-fibrotic action. This statement finds further support when we consider the statistically significant interaction between P3NP and leptin. Indeed, we know that leptin is an adipose tissue-derived hormone [25] that has a pro-fibrotic action at the hepatic, renal, and myocardial levels; regulates skeletal muscle metabolism; and acts as a pro-angiogenic factor [25]. Leptin-induced fibrotic response is a complex process that involves multiple cells and is regulated by multiple molecular pathway (reviewed in Mao) [26]. Thus, the interaction of leptin with P3NP indicates that a fibrotic process is activated [27]. With available data from the InCHIANTI study, we are unable to demonstrate where this process is active, which pathways could be activated, and those aspects could be the subject of further experimental studies.

On the other hand, the statistically significant interaction (P3NP for muscle density) could be a biomarker for muscle remodeling process. It is important to highlight that intramuscular connective tissue (IMCT) wraps muscle fibers, and both are part of the functional muscle–tendon-bone unit [28]. IMCT plays a crucial role in muscle force transmission [29] and motor coordination and contributes to the elastic tissue response of the muscle [30]. IMCT consists of two components: cells and the extracellular matrix (ECM) [31]. The ECM is mostly composed of collagen, elastin, and an amorphous phase rich in glycoproteins and hyaluronan. During aging, our muscles and tendons increase in stiffness because of tissue changes, including collagen accumulation. The proportion of muscle area occupied by ECM also increases, and elderly subjects have about 2.5 times more ECM than young subjects [32]. In summary, the relationship between P3NP and muscle density may indicate both pro-fibrotic action and muscle remodeling.

From a pathogenic perspective, the differentiation of fibro-adipogenic progenitor (FAP) cells towards a fibrogenic state may result in increased intramuscular fibrosis during the aging process [31]. In an experimental mouse model, a significant proinflammatory stimulus contributed to the differentiation of FAP cells into adipocytes leading to an extensive adipose infiltrate (myosteatosis) [33]. In general, the infiltration of muscular and fascia structures by adipose and/or fibrotic tissue could explain the reduction of muscle density observed in the InCHIANTI population study.

Finally, our research has confirmed the potential role of P3NP as a marker of fibrosis in various diseases, including chronic liver conditions, CHF, hypertension, and atherosclerosis [34]. The suggested role of P3NP might be primarily attributed to structural and functional remodeling of the arterial wall, such as intimal medial thickening (IMT) and stiffening [35]. The age-related increase in collagen deposition within the arterial wall is known as arterial profibrosis [34], and is associated with a systemic pro-inflammatory status that occurs with aging [36]. Moreover, our data indicate that P3NP might be a biomarker predicting OA, a suggestive new hypothesis which has not been investigated so far. It has been hypothesized that myofascial fibrosis, due to the imbalance between the ratio of collagen-I and collagen-III, might contribute to symptomatic OA [37], and our findings show a possible indicator for this condition.

### Limitations

A relevant limitation of our study concerns the consistency of P3NP measurement over time. The plasma level of this biomarker was only assessed at a single time point (baseline), and it is possible that longitudinal variations could provide a more accurate measure of the association between aging and diffuse fibrosis. In addition, P3NP was assessed by venous sampling, which does not necessarily represent its action at the local muscle level, but a much broader, and systemic phenomenon; in fact, raise of P3NP serum levels could represent a primitive fibrosis damage of other organs and apparatus (e.g., cardiovascular, hepatic, and renal systems). Finally due to the epidemiological study design, we were unable to explain potential underlying mechanism for reported associations.

## Conclusion

Our long-term observational study on a free-living large population of individuals indicates that high serum P3NP is a biomarker of the development of fibrotic processes related to new disease manifestations, subsequent to the increase in the comorbidities; P3NP is also related to skeletal muscle density alterations, particularly in terms of fatty infiltration or diffuse fibrosis, and correlates with poor physical performance during aging.

### Supplementary Information

Below is the link to the electronic supplementary material.

Supplementary file1 (DOCX 711 KB)

## Data Availability

The datasets used and/or analyzed during the current study are available from the responsible authors for the InCHIANTI study (Luigi Ferrucci) on reasonable request. Data of the InCHIANTI study is available to all researchers upon justified request using the proposal form available on the InChianti website (https://www.nia.nih.gov/inchianti-study, accessed on April 13, 2023).

## References

[1] Costanzo L, De Vincentis A, Di Iorio A, Bandinelli S, Ferrucci L, Antonelli Incalzi R (2020). Impact of low muscle mass and low muscle strength according to EWGSOP2 and EWGSOP1 in community-dwelling older people. J Gerontol A Biol Sci Med Sci.

[2] Correa-De-Araujo R, Hadley E (2014). Editor’s choice: Skeletal muscle function deficit: A new terminology to embrace the evolving concepts of sarcopenia and age-related muscle dysfunction. J Gerontol Ser A Biol Sci Med Sci.

[3] Pinel S, Kelp NY, Bugeja JM, Bolsterlee B, Hug F, Dick TJM. Quantity versus quality: Age-related differences in muscle volume, intramuscular fat, and mechanical properties in the triceps surae. Exp Gerontol. 2021; 156. 10.1016/J.EXGER.2021.111594 10.1016/j.exger.2021.111594 34673171

[4] Miljkovic I, Zmuda JM (2010). Epidemiology of myosteatosis. Curr Opin Clin Nutr Metab Care.

[5] Sinanan ACM, Buxton PG, Lewis MP (2006). Muscling in on stem cells. Biol cell.

[6] Eshima H (2021). Influence of obesity and type 2 diabetes on calcium handling by skeletal muscle: Spotlight on the sarcoplasmic reticulum and mitochondria. Front Physiol.

[7] Correa-de-Araujo R, Harris-Love MO, Miljkovic I, Fragala MS, Anthony BW, Manini TM. The Need for standardized assessment of muscle quality in skeletal muscle function deficit and other aging-related muscle dysfunctions: A symposium report. Front Physiol. 2017; 8. 10.3389/FPHYS.2017.00087 10.3389/fphys.2017.00087 PMC5310167 28261109

[8] Wang X, Zhou L. The many roles of macrophages in skeletal muscle injury and repair. Front cell Dev Biol. 2022; 10. 10.3389/FCELL.2022.952249 10.3389/fcell.2022.952249 PMC9309511 35898401

[9] Zoico E, Corzato F, Bambace C, Rossi AP, Micciolo R, Cinti S (2013). Myosteatosis and myofibrosis: Relationship with aging, inflammation and insulin resistance. Arch Gerontol Geriatr.

[10] Chen YY, Chiu YL, Kao TW, Peng TC, Yang HF, Chen WL. Cross-sectional associations among P3NP, HtrA, Hsp70, Apelin and sarcopenia in Taiwanese population. BMC Geriatr. 2021; 21. 10.1186/S12877-021-02146-5 10.1186/s12877-021-02146-5 PMC7980650 33743591

[11] Santanasto AJ, Cvejkus RK, Wojczynski MK, Marron MM, Schupf N, Christensen K (2021). Circulating procollagen type III N-terminal peptide and physical function in adults from the long life family study. J Gerontol A Biol Sci Med Sci.

[12] Shin HE, Kim M, Won CW. Association between plasma procollagen type III N-terminal peptide (P3NP) levels and physical performance in elderly men: The Korean Frailty and Aging Cohort Study (KFACS). Exp Gerontol. 2021; 154. 10.1016/j.exger.2021.111523 10.1016/j.exger.2021.111523 34425203

[13] López B, González A, Díez J (2010). Circulating biomarkers of collagen metabolism in cardiac diseases. Circulation.

[14] Curcio F, Ferro G, Basile C, Liguori I, Parrella P, Pirozzi F (2016). Biomarkers in sarcopenia: A multifactorial approach. Exp Gerontol.

[15] Ferrucci L, Bandinelli S, Benvenuti E, Di Iorio A, Macchi C, Harris TB (2000). Subsystems contributing to the decline in ability to walk: Bridging the gap between epidemiology and geriatric practice in the InCHIANTI study. J Am Geriatr Soc.

[16] Russo CR, Lauretani F, Bandinelli S, Bartali B, Di Iorio A, Volpato S, et al. Aging bone in men and women: Beyond changes in bone mineral density. Osteoporos Int. 2003; 14. 10.1007/s00198-002-1322-y 10.1007/s00198-002-1322-y 12827220

[17] Rowe GS, Blazevich AJ, Haff GG (2019). pQCT- and ultrasound-based muscle and fat estimate errors after resistance exercise. Med Sci Sports Exerc.

[18] Simonsick EM, Maffeo CE, Rogers SK, Skinner EA, Davis D, Guralnik JM, et al. Methodology and feasibility of a home-based examination in disabled older women: The Women’s Health and Aging Study. J Gerontol A Biol Sci Med Sci. 1997; 52. 10.1093/GERONA/52A.5.M264 10.1093/gerona/52a.5.m264 9310080

[19] Guralnik JM, Ferrucci L, Simonsick EM, Salive ME, Wallace RB (1995). Lower-extremity function in persons over the age of 70 years as a predictor of subsequent disability. N Engl J Med.

[20] Zou G (2004). A modified poisson regression approach to prospective studies with binary data. Am J Epidemiol.

[21] Berry SD, Ramachandran VS, Cawthon PM, Gona P, McLean RR, Cupples LA, et al. Procollagen type III N-terminal peptide (P3NP) and lean mass: A cross-sectional study. J frailty aging. 2013; 2: 129–134. Im Internet: http://www.ncbi.nlm.nih.gov/pubmed/24244927. Stand: 27.11.2022 PMC3828748 24244927

[22] Kruszewska J, Cudnoch-Jedrzejewska A, Czarzasta K. Remodeling and fibrosis of the cardiac muscle in the course of obesity-pathogenesis and involvement of the extracellular matrix. Int J Mol Sci. 2022; 23. 10.3390/IJMS23084195 10.3390/ijms23084195 PMC9032681 35457013

[23] GazotiDebessa CR, MesianoMaifrino LB, Rodrigues de Souza R (2001). Age related changes of the collagen network of the human heart. Mech Ageing Dev.

[24] López B, Ravassa S, Moreno MU, José GS, Beaumont J, González A (2021). Diffuse myocardial fibrosis: Mechanisms, diagnosis and therapeutic approaches. Nat Rev Cardiol.

[25] Nwadozi E, Ng A, Strömberg A, Liu Hyi, Olsson K, Gustafsson T (2019). Leptin is a physiological regulator of skeletal muscle angiogenesis and is locally produced by PDGFRα and PDGFRβ expressing perivascular cells. Angiogenesis.

[26] Mao Y, Zhao K, Li P, Sheng Y (2023). The emerging role of leptin in obesity-associated cardiac fibrosis: Evidence and mechanism. Mol Cell Biochem.

[27] Madani S, De Girolamo S, Muñoz DM, Li RK, Sweeney G (2006). Direct effects of leptin on size and extracellular matrix components of human pediatric ventricular myocytes. Cardiovasc Res.

[28] Blottner D, Huang Y, Trautmann G, Sun L (2019). The fascia: Continuum linking bone and myofascial bag for global and local body movement control on Earth and in Space. A scoping review. REACH.

[29] Turrina A, Martínez-González MA, Stecco C (2013). The muscular force transmission system: Role of the intramuscular connective tissue. J Bodyw Mov Ther.

[30] Huijing PA (2009). Epimuscular myofascial force transmission: A historical review and implications for new research. International Society of Biomechanics Muybridge Award Lecture, Taipei, 2007. J Biomech.

[31] Fede C, Fan C, Pirri C, Petrelli L, Biz C, Porzionato A, et al. The effects of aging on the intramuscular connective tissue. Int J Mol Sci. 2022; 23. 10.3390/IJMS231911061 10.3390/ijms231911061 PMC9569538 36232366

[32] Pavan P, Monti E, Bondí M, Fan C, Stecco C, Narici M, et al. Alterations of extracellular matrix mechanical properties contribute to age-related functional impairment of human skeletal muscles. Int J Mol Sci. 2020; 21. 10.3390/IJMS21113992 10.3390/ijms21113992 PMC7312402 32498422

[33] Giuliani G, Rosina M, Reggio A (2022). Signaling pathways regulating the fate of fibro/adipogenic progenitors (FAPs) in skeletal muscle regeneration and disease. FEBS J.

[34] Wang M, Monticone RE, McGraw KR (2020). Proinflammation, profibrosis, and arterial aging. Aging Med milt.

[35] Di Iorio A, Di Blasio A, Napolitano G, Ripari P, Paganelli R, Cipollone F (2019). High fat mass, low muscle mass, and arterial stiffness in a population of free-living healthy subjects: The "al passo con la tua salute" project. Medicine (Baltimore).

[36] Frasca D, Blomberg BB, Paganelli R. Aging, obesity, and inflammatory age-related diseases. Front Immunol. 2017; 8. 10.3389/fimmu.2017.01745 10.3389/fimmu.2017.01745 PMC5725402 29270179

[37] Fantoni I, Biz C, Fan C, Pirri C, Fede C, Petrelli L, et al. Fascia lata alterations in hip osteoarthritis: An observational cross-sectional study. Life (Basel, Switzerland). 2021; 11. 10.3390/LIFE11111136 10.3390/life11111136 PMC8625990 34833012

