# Supplementary material for: Muscle quality, physical performance, and comorbidity are predicted by circulating procollagen type III N-terminal peptide (P3NP): the InCHIANTI follow-up study
Source: GeroScience. 2023 Aug 2;46(1):1259–69. doi: 10.1007/s11357-023-00894-3 (PMC10828316; doi:10.1007/s11357-023-00894-3)
Supplement: Supplementary file 1 — Supplementary file1 (DOCX 711 KB) [file 11357_2023_894_MOESM1_ESM.docx]

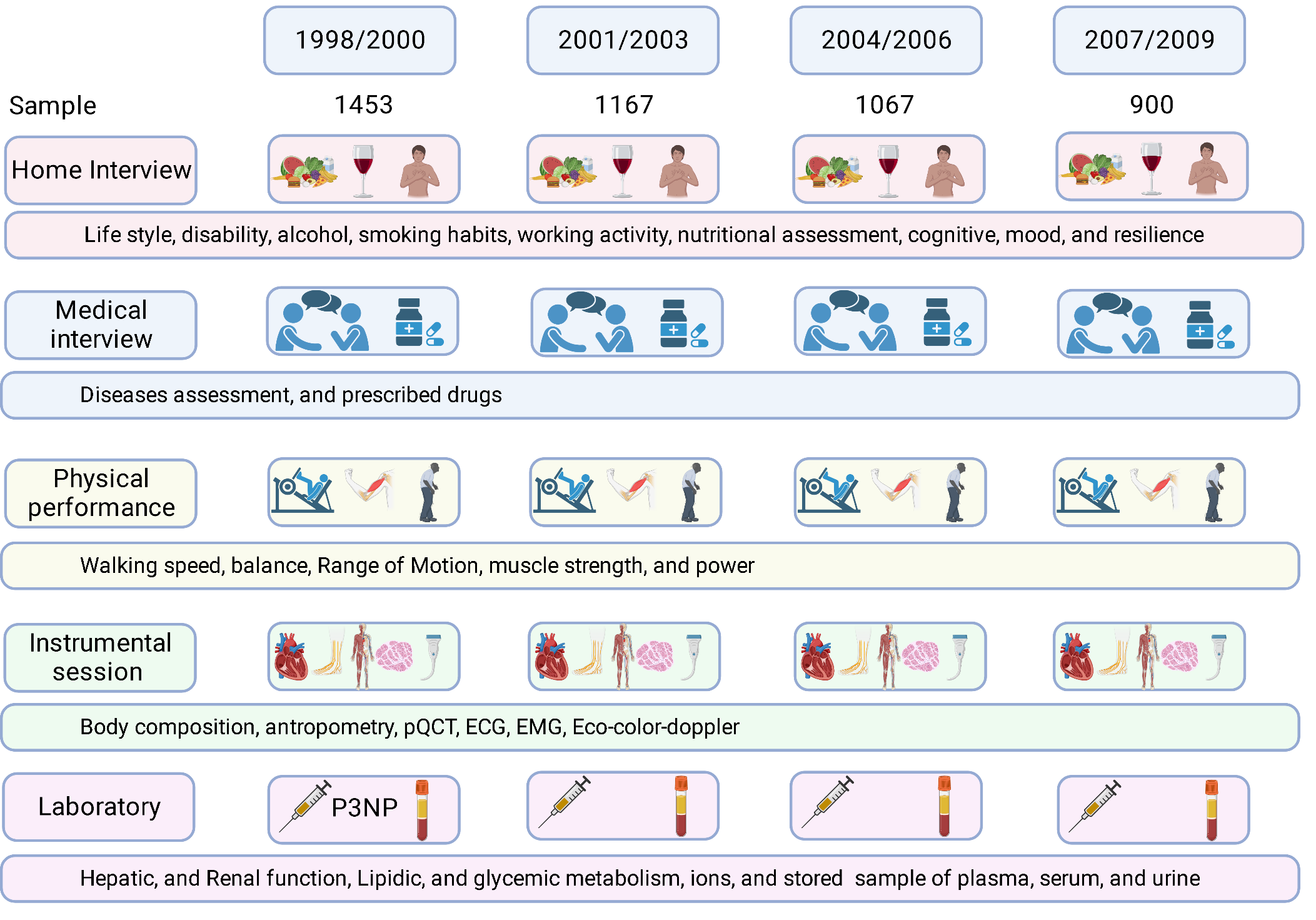


Supplementary Figure 1: The InCHIANTI study design. Figure is created with Biorender ([www.biorender.com](http://www.biorender.com), Toronto ON M5V 2J1 Canada) licensed to ADI.

InCHIANTI (**In**vecchiare in **Chianti**, aging in the Chianti area) is a study of the factors contributing to the decline of mobility in late life conducted in two small town in the countryside of the Tuscany area (*Greve in Chianti* and *Bagno a Ripoli*) (1). The data collection started in September 1998 and was completed in March 2000. In August 1998, were randomly selected from the population registry of the two sites 1453 persons. After receiving a letter of invitation, a home interview was scheduled by telephone.

**Home interview** collected information on home barriers, family composition and social networks, cognitive and depressive symptoms, ability to perform daily life activities, foot problems, falls and fear of falling, incontinence, quality of sleep, and food intake.

**Instrumental session** include: a peripheral quantitative computed tomography, a surface electroneurography, a standard electrocardiogram, an ultrasound color doppler examination of the carotids, vertebral arteries and veins of the lower limbs, and an assessment of the ankle-brachial index; blood sample was drawn and were stored; moreover, was also stored an aliquot of 24-hour urine collection. Procollagen Type III N-Terminal Peptide (P3NP) was assessed only at baseline (1998-2000).

**Medical Interview:** a trained geriatricians explore and assessed major medical conditions and drugs assumptions.

**Physical performance**: was aimed at objectively assessing physical function, muscle and power strength, joint range of motion and walking abilities.

The study design of the InCHIANTI follow-up conduct every three-year, repeated the same measures that were performed at baseline.

([InCHIANTI Study | National Institute on Aging (nih.gov)](https://www.nia.nih.gov/inchianti-study#design)).

1. Ferrucci L, Bandinelli S, Benvenuti E, et al. Subsystems contributing to the decline in ability to walk: Bridging the gap between epidemiology and geriatric practice in the InCHIANTI study. *J Am Geriatr Soc*. 2000;48(12):1618-1625. doi:10.1111/j.1532-5415.2000.tb03873.x.
